# Subcortical Circuits Among Pedunculopontine Nucleus, Thalamus and Basal Ganglia Play Important Roles in Paroxysmal Arousal in Genetic Rat Models of Autosomal Dominant Sleep-Related Hypermotor Epilepsy

**DOI:** 10.3390/ijms26125522

**Published:** 2025-06-09

**Authors:** Ruri Okubo, Eishi Motomura, Motohiro Okada

**Affiliations:** Department of Neuropsychiatry, Division of Neuroscience, Graduate School of Medicine, Mie University, Tsu 514-8507, Japan; okubo-r@med.mie-u.ac.jp (R.O.); motomura@clin.medic.mie-u.ac.jp (E.M.)

**Keywords:** ADSHE, tripartite synaptic transmission, glutamate, ACh, hemichannel

## Abstract

A part of autosomal dominant sleep-related hypermotor epilepsy (ADSHE) is caused by mutant CHRNA4. The pathomechanisms underlying motor seizures followingly brief/sudden awakening (paroxysmal arousal) in ADSHE seizures remain to be clarified. This study determined extracellular levels of ACh and L-glutamate in the pedunculopontine nucleus (PPN) and its projection regions, including the thalamus and basal ganglia, during wakefulness, slow-wave sleep (SWS) and paroxysmal arousal of transgenic rats bearing rat S286L-mutant Chrna4 (S286L-TG), corresponding to human S284L-mutant CHRNA4, using microdialysis. The expression of connexin43 and pannexin1 in the plasma membrane of the PPN was determined using capillary immunoblotting. The expressions of connexin43 and pannexin1 in the PPN plasma membrane of S286L-TG were larger than the wild type. The extracellular L-glutamate levels in the PPN and projection regions of S286L-TG consistently increased during both wakefulness and SWS compared to the wild type. The extracellular levels of ACh and L-glutamate in the PPN and projection regions decreased accompaning SWS in the wild type. In S286L-TG, this decreasing extracellular ACh level was observed, whereas decreasing L-glutamate level was impaired. Both extracellular levels of ACh and L-glutamate in the PPN and projection regions drastically increased during paroxysmal arousal. Hemichannel inhibitors suppressed the increasing releases of ACh and L-glutamate induced by paroxysmal arousal but decreased and did not affect extracellular levels of L-glutamate and ACh during wakefulness and SWS, respectively. In particular, under hemichannels inhibition, decreasing L-glutamate release accompanying SWS was observed in S286L-TG. This study elucidated that enhanced hemichannels are predominantly involved in the dysfunction of glutamatergic transmission compared to AChergic transmission during the interictal stage in S286L-TG, whereas the hyperactivation of hemichannels contributes to the generation of paroxysmal arousal. Therefore, the hyperactivated excitatory tripartite synaptic transmission associated with hemichannels in the PPN and projection regions plays important roles in epileptogenesis/ictogenesis in S286L-TG.

## 1. Introduction

Various mutations of genes encoding subtypes of nicotinic acetylcholine (ACh) receptors (nAChRs) and others have been identified in individuals/pedigrees with autosomal dominant (ADSHE) or sporadic forms (SHE) of sleep-related hypermotor epilepsies (previously ADNFLE/NFLE) [1,2,3,4,5,6]. Typical features of ADSHE/SHE seizures are minormotor events and hypermotor events, including hyperkinetic automatisms, axial/pelvic movements and asymmetric tonic/dystonic posturing following brief/sudden awakenings during slow-wave sleep (SWS); however, epileptic discharges during ADSHE/SHE seizures are not always detectable in electroencephalograms (EEG) [3,7,8,9]. We have already detected epileptic seizures resembling ADSHE/SHE seizures in genetic ADSHE rat models, so-called “S286L-TG” and “S284L-TG”, bearing rat missense S286L-mutant *Chrna4*, which corresponds to the S284L mutation in human *CHRNA4* [1,10,11]. Several complicated epileptogenesis/ictogenesis features of S286L-TG were also reported [1,10,11,12]. Loss-of-function S284L-mutant nAChR [13,14,15] generates GABAergic disinhibition, leading to enhanced propagation of high-frequency oscillation (HFO) during SWS to various regions, including the thalamus, cortex and basal ganglia [1,10,11,16]. Enhanced excitabilities reinforce astroglial excitatory tripartite synaptic transmissions via age-dependently increasing expression and function of the astroglial hemichannels [1,12,16,17,18]. It is well known that hemichannels, which are mainly composed connexin43 and pannexin1, are the major molecules responsible for non-exocytotic astroglial transmitter releases in tripartite synaptic transmission, and hyperactivated hemichannels increase the extracellular levels of excitatory transmitters, such as L-glutamate, D-serine and ATP [1,17,18,19,20]. Therefore, synergic interactions among hyperactivated astroglial hemichannels, GABAergic disinhibition and physiological HFO possibly play fundamental roles in the development of epileptogenesis/ictogenesis in ADSHE [1,12].

**Figure 1:**
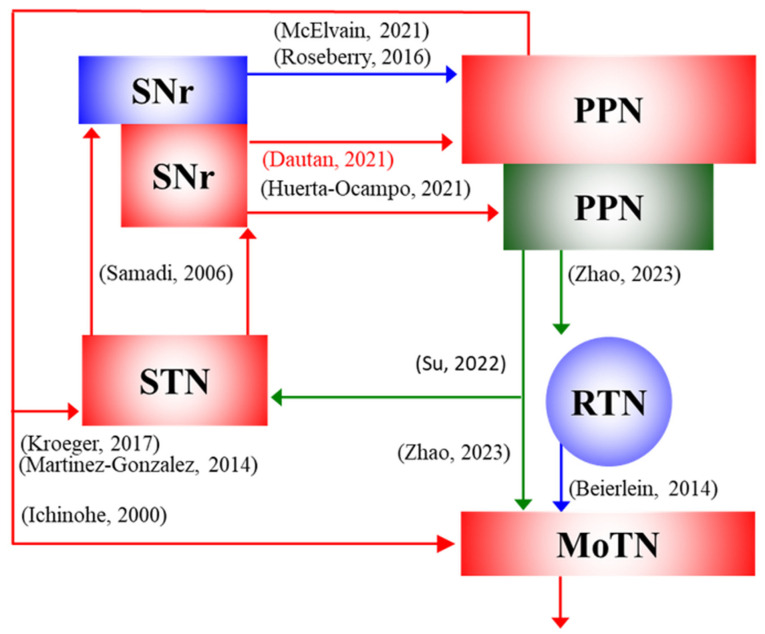
Based on the established networks, the present study was designed to determine the transmission abnormalities in the AChergic and glutamatergic projections from the pedunculopontine nucleus (PPN) to its projection regions, such as the thalamus (RTN and MoTN) and subthalamic nucleus (STN) [21,22,23,24,25,26], and afferents from the substantia nigra reticulata (SNr) to the PPN [27,28,29,30,31].

ADSHE phenotypes of both S284L-TG and S286L-TG were evaluated to be milder compared to most untreated ADSHE patients, since the frequency of hypermotor seizures was approximately once a week, without clustering [1,11,32,33]. In our previous studies, polyspikes lasting several seconds without accompanying movements were counted as interictal discharges [1,10,33,34]; however, eye opening during polyspikes was observed, which is a similar phenotype to clinically characterized paroxysmal arousals [3,8,9]. Indeed, polyspikes frequencies were decreased by zonisamide and benzodiazepine, but not by carbamazepine [10,11,33], similar to ADSHE patients with the S284L mutation [1,9,35,36,37]. Paroxysmal arousals are obviously distinct seizures from other focal seizures (limbic seizures are generally accompanied by unconsciousness) [3,8,9,38]. Therefore, identifying transmission abnormalities during eye opening with polyspikes as paroxysmal arousal should contribute to a further understanding of the epileptogenesis/ictogenesis of ADSHE. Mechanisms by which subcortical networks regulate the sleep–wake cycle and locomotor initiation have traditionally been explored, with evidence pointing to the pedunculopontine nucleus (PPN), which projects AChergic and glutamatergic terminals, as a crucial node [39,40,41,42]. Recently, intensive studies have been elucidating networks among the PPN, thalamus and basal ganglia for regulation of the sleep–wake cycle and locomotion. Both AChergic and glutamatergic neurons in the PPN project to the thalamus and subthalamic nucleus (STN) and receive GABAergic and glutamatergic terminals from the substantia nigra reticulata (SNr) (Figure 1) [10,11,16,21,22,23,24,26,27,28,29,31].

Considering the previous findings, exploring the mechanism of attenuated AChergic transmission and enhanced glutamatergic transmission in networks among the PPN, thalamus and basal ganglia (STN and SNr) due to loss-of-function S284L-mutant nAChRs can provide important insights into the epileptogenesis/ictogenesis of ADSHE with S284L mutation. Therefore, based on these hypotheses, this study determined extracellular levels of ACh, GABA and L-glutamate in the PPN, thalamus, STN and SNr during wakefulness, SWS and arousal with polyspikes (as candidate paroxysmal arousal) of S286L-TG using in vivo microdialysis, which has been established as an experimental method for collecting extracellular transmitters [43].

## 2. Results

### 2.1. Extracellular Levels of ACh, L-Glutamate and GABA During Wakefulness, SWS and Interictal Discharge, and Expression of Connexin43 and Pannexin1 in the PPN

**Figure 2:**
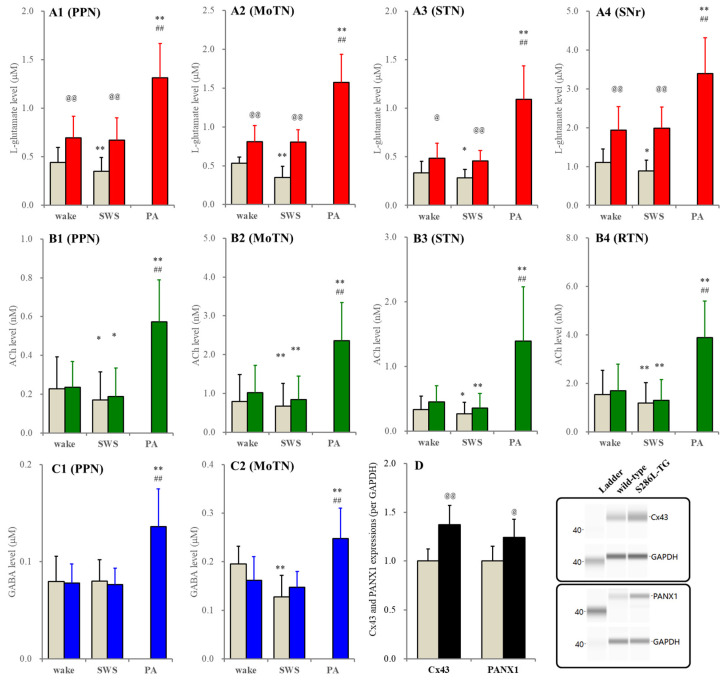
Panels (**A**–**C**) indicate fluctuations of extracellular levels of L-glutamate, ACh and GABA of the wild type (grey columns) and S286L-TG (red, green and blue columns) during wakefulness (wake), SWS and paroxysmal arousal (PA) in the PPN (**A1**–**C1**), MoTN (**A2**–**C2**), STN (**A3**–**B3**), SNr (**A4**) and RTN (**B4**), respectively. Ordinates indicate the mean ± SD (*n* = 6) of extracellular levels of ACh (nM), L-glutamate (μM) and GABA (μM). Panel (**D**) indicates the expression of connexin43 (Cx43) and pannexin1 (PANX1) in the plasma membrane fraction of the PPN between wild type (grey columns) and S286L-TG (black columns). Ordinates indicate the mean ± SD (n = 6) of the relative expression of connexin43 and pannexin1 per GAPDH. Right-side panels indicate the pseudo-gel images of connexin43, pannexin1 and GAPDH, obtained using capillary immunoblotting. * *p* < 0.05, ** *p* < 0.01 relative to wakefulness, @ *p* < 0.05, @@ *p* < 0.01 relative to wild type ## *p* < 0.01 relative to SWS using MANOVA with Scheffe’s post hoc test or Student’s *T*-test. F-values of L-glutamate between wakefulness and SWS were in the PPN [F_genotype_(1,10) = 7.0 (*p* < 0.05), F_event_(1,10) = 7.2 (*p* < 0.05), F_genotype*event_(1,10) = 2.6 (*p* > 0.1)], MoTN [F_genotype_(1,10) = 17.4 (*p* < 0.01), F_event_(1,10) = 27.1 (*p* < 0.01), F_genotype*event_(1,10) = 25.3 (*p* < 0.01)], STN [F_genotype_(1,10) = 5.8 (*p* < 0.05), F_event_(1,10) = 5.7 (*p* < 0.05), F_genotype*event_(1,10) = 0.5 (*p* > 0.1)] and SNr [F_genotype_(1,10) = 13.8 (*p* < 0.05), F_event_(1,10) = 1.9 (*p* > 0.1), F_genotype*event_(1,10) = 5.4 (*p* < 0.05)]. F-values of ACh between wakefulness and SWS were in the PPN [F_genotype_(1,10) = 0.1 (*p* > 0.1), F_event_(1,10) = 9.2 (*p* < 0.05), F_genotype*event_(1,10) = 0.1 (*p* > 0.1)], MoTN [F_genotype_(1,10) = 0.3 (*p* > 0.1), F_event_(1,10) = 13.2 (*p* < 0.01), F_genotype*event_(1,10) = 0.4 (*p* > 0.1)], STN [F_genotype_(1,10) = 0.7 (*p* > 0.1), F_event_(1,10) = 23.0 (*p* < 0.01), F_genotype*event_(1,10) = 0.5 (*p* > 0.1)] and RTN [F_genotype_(1,10) = 0.1 (*p* > 0.1), F_event_(1,10) = 21.9 (*p* < 0.01), F_genotype*event_(1,10) = 0.1 (*p* > 0.1)]. F-values of GABA between wakefulness and SWS were in the PPN [F_genotype_(1,10) = 0.1 (*p* > 0.1), F_event_(1,10) = 0.1 (*p* > 0.1), F_genotype*event_(1,10) = 0.2 (*p* > 0.1)] and MoTN [F_genotype_(1,10) = 0.3 (*p* > 0.1), F_event_(1,10) = 31.4 (*p* < 0.01), F_genotype*event_(1,10) = 13.0 (*p* < 0.01)]. F-values of L-glutamate among wakefulness, SWS and IID of S286L-TG were in the PPN [F(2,10) = 67.4 (*p* < 0.01)], MoTN [F(1.0,5.2) = 83.9 (*p* < 0.01)], STN [F(1.1,5.3) = 45.9 (*p* < 0.01)] and SNr [F(2,10) = 76.5 (*p* < 0.01)]. F-values of ACh among wakefulness, SWS and interictal discharge of S286L-TG were in the PPN [F(2,10) = 43.7 (*p* < 0.01)], MoTN [F(1.1,5.5) = 39.4 (*p* < 0.01)], STN [F(1.0,5.1) = 14.8 (*p* < 0.01)] and RTN [F(2,10) = 51.2 (*p* < 0.01]. F-values of GABA among wakefulness, SWS and IID of S286L-TG were in the PPN [F(2,10) = 34.7 (*p* < 0.01)] and MoTN [F(2,10) = 53.4 (*p* < 0.01)].

In the wild type, extracellular L-glutamate levels in the PPN, MoTN, STN and SNr during SWS decreased compared to wakefulness, whereas those reductions were abolished in S286L-TG (Figure 2). Furthermore, extracellular L-glutamate levels during both wakefulness and SWS in the PPN, MoTN, STN and SNr of S286L-TG were consistently larger than the wild type (Figure 2). However, extracellular GABA levels in the PPN during wakefulness and SWS between the wild type and S286L-TG were almost equal. In the MoTN, the extracellular GABA level during SWS in the wild type decreased, but that in S286L-TG was unchanged compared to wakefulness. In the comparison between the wild type and S286L-TG, significant differences in the extracellular GABA level during both wakefulness and SWS could not be detected (Figure 2).

**Figure 3:**
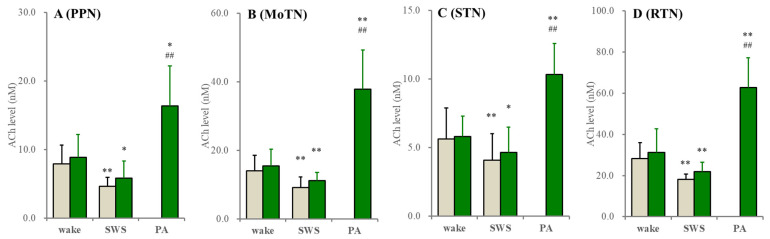
Fluctuations of extracellular ACh levels during wakefulness (wake), SWS and paroxysmal arousal in the PPN, MoTN, STN and RTN. Fluctuations of extracellular ACh levels of the wild type (grey columns) and S286L-TG (green columns) during wakefulness, SWS and paroxysmal arousal (PA) in the PPN (**A**), MoTN (**B**), STN (**C**) and RTN (**D**) under the perfusion of MRS containing 1 μM neostigmine. Ordinates indicate the mean ± SD (*n* = 6) of extracellular ACh level (nM). * *p* < 0.05, ** *p* < 0.01 relative to wakefulness ## *p* < 0.01 relative to SWS using MANOVA with Scheffe’s post hoc test. F-values between wakefulness and SWS were in the PPN [F_genotype_(1,10) = 0.6 (*p* > 0.1), F_event_(1,10) = 69.2 (*p* < 0.01), F_genotype*event_(1,10) = 0.1 (*p* > 0.1)], MoTN [F_genotype_(1,10) = 0.7 (*p* > 0.1), F_event_(1,10) = 44.4 (*p* < 0.01), F_genotype*event_(1,10) = 0.3 (*p* > 0.1)], STN [F_genotype_(1,10) = 0.2 (*p* > 0.1), F_event_(1,10) = 7.7 (*p* < 0.05), F_genotype*event_(1,10) = 0.2 (*p* > 0.1)] and RTN [F_genotype_(1,10) = 0.8 (*p* > 0.1), F_event_(1,10) = 20.7 (*p* < 0.01), F_genotype*event_(1,10) = 0.1 (*p* > 0.1)]. F-values of ACh among wakefulness, SWS and interictal discharge of S286L-TG were in the PPN [F(1.1,5.5) = 41.2 (*p* < 0.01)], MoTN [F(1.1,5.5) = 40.8 (*p* < 0.01)], STN [F(1.0,5.1) = 50.2 (*p* < 0.01)] and RTN [F(2,10) = 67.9 (*p* < 0.01].

In both the wild type and S286L-TG, extracellular ACh levels in the PPN, MoTN, STN and RTN during SWS decreased compared to wakefulness. Extracellular ACh levels were almost equivalent between the wild type and S286L-TG (Figure 2). It is well known that the collected ACh level in the perfusate using microdialysis is very low due to being rapidly degraded by acetylcholinesterase/butyrylcholinesterase in the synaptic cleft [44,45,46]. Therefore, the fluctuations of extracellular ACh levels were also determined using the conventional method, perfused MRS containing 1 μM neostigmine. When MRS containing neostigmine was perfused into the PPN, MoTN, STN or RTN, the extracellular ACh levels in these regions between the wild type and S286L-TG were almost equal, and their decreasing during SWS compared to wakefulness was also detectable (Figure 3).

**Figure 4:**
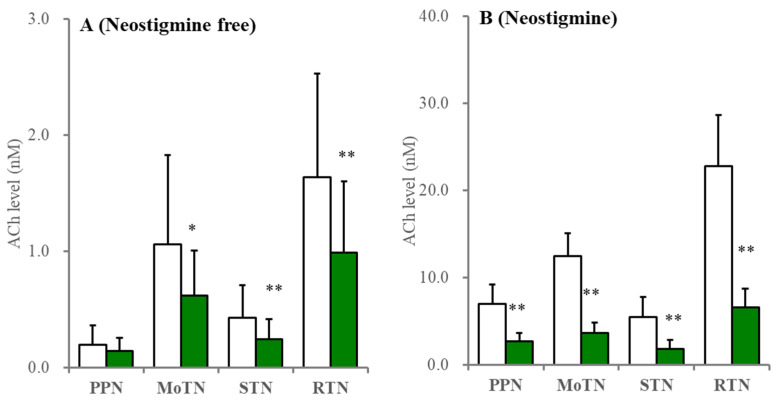
Effects of perfusion with tetrodotoxin on extracellular ACh levels in the PPN, MoTN, STN and RTN. Under the perfusion of MRS containing without (**A**) or with (**B**) 1 μM neostigmine, the effects of perfusion with 1 μM TTX (green columns) into the PPN, MoTN, STN and RTN on extracellular ACh level in the PPN, MoTN, STN and RTN, respectively. Ordinates indicate the mean ± SD (*n* = 6) of extracellular ACh level (nM). * *p* < 0.05, ** *p* < 0.01 relative to control (TTX free) using Student’s *T*-test.

Furthermore, perfusion with 1 μM tetrodotoxin decreased ACh levels in the RTN, MoTN and STN by approximately 80% and, in the PPN, by approximately 60%, when MRS containing neostigmine was perfused; however, tetrodotoxin decreased ACh levels in the RTN, MoTN and STN by approximately 50% but did not affect the PPN when neostigmine-free MRS was perfused (Figure 4).

These results suggest that functional abnormalities of acetylcholinesterase/butyrylcholinesterase in S286L-TG are unlikely to contribute to the ictogenesis/epileptogenesis of S286L-TG. Extracellular levels of L-glutamate, ACh and GABA during paroxysmal arousal (eye opening with polyspikes) in all experimental regions of S286L-TG increased compared to wakefulness and SWS (Figure 2 and Figure 3). In the PPN, expressions of both connexin43 and pannexin1 in the PPN plasma membrane of S286L-TG increased compared to the wild type (Figure 2), resembling the frontal cortex and thalamus [16,18]. Therefore, there are possibly no functional abnormalities in acetylcholinesterase/butyrylcholinesterase in S286L-TG, whereas the functional abnormalities in the exocytosis of ACh (impaired ACh release) probably play important roles in the pathomechanisms of ADSHE in S286L-TG.

### 2.2. Effects of Perfusion with Hemichannel Inhibitors into the PPN on Extracellular Levels of ACh and L-Glutamate During Wakefulness, SWS and Interictal Discharge

**Figure 5:**
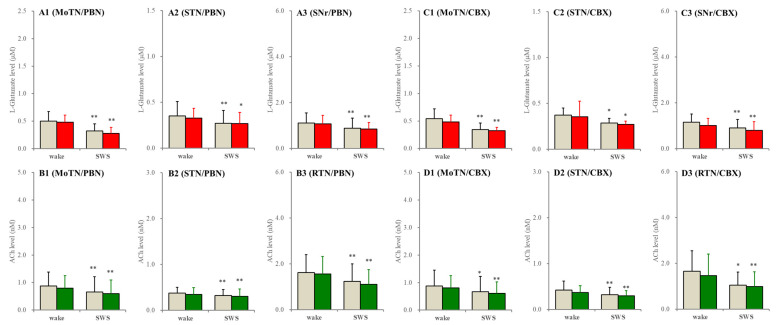
Effects of perfusion with hemichannel inhibitors into the PPN on extracellular levels of L-glutamate and ACh during wakefulness (wake) and SWS in the MoTN, STN, SNr and RTN of the wild type. Panels (**A**,**B**) and (**C**,**D**) indicate the effects of perfusion with 300 μM probenecid (pannexin1-hemichannel inhibitor) and 100 μM carbenoxolone (non-selective connexin43-hemichannel inhibitor) on extracellular levels of L-glutamate (panels **A** and **C**) and ACh (panels **B** and **D**) in the MoTN (panels **A1**–**D1**), STN (panels **A2**–**D2**), SNr (panels **A3** and **C3**) and RTN (panels **B3** and **D3**) of the wild type during wakefulness and SWS. Ordinates indicate the mean ± SD (*n* = 6) of extracellular levels of ACh (nM) and L-glutamate (μM). Grey columns indicate the levels of control (perfusion with MRS alone). Red and green columns indicate the levels of L-glutamate and ACh during the perfusion with MRS containing probenecid or carbenoxolone, respectively. * *p* < 0.05, ** *p* < 0.01 relative to wakefulness using MANOVA with Scheffe’s post hoc test. F-values of effects of probenecid on L-glutamate level in the MoTN [F_PBN_(1,5) = 1.9 (*p* > 0.1), F_event_(1,5) = 85.2 (*p* < 0.01), F_PBN*event_(1,5) = 0.4 (*p* > 0.1)], STN [F_PBN_(1,5) = 0.3 (*p* > 0.1), F_event_(1,5) = 9.1 (*p* < 0.01), F_PBN*event_(1,5) = 0.2 (*p* > 0.1)] and SNr [F_PBN_(1,5) = 0.3 (*p* > 0.1), F_event_(1,5) = 37.5 (*p* < 0.01), F_PBN*event_(1,5) = 0.1 (*p* > 0.1)]. F-values of effects of carbenoxolone on L-glutamate level in the MoTN [F_CBX_(1,5) = 0.5 (*p* > 0.1), F_event_(1,5) = 30.1 (*p* < 0.01), F_CBX*event_(1,5) = 0.1 (*p* > 0.1)], STN [F_CBX_(1,5) = 0.1 (*p* > 0.1), F_event_(1,5) = 6.7 (*p* < 0.05), F_CBX*event_(1,5) = 0.1 (*p* > 0.1)] and SNr [F_CBX_(1,5) = 3.5 (*p* > 0.1), F_event_(1,5) = 21.3 (*p* < 0.01), F_CBX*event_(1,5) = 0.1 (*p* > 0.1)]. F-values of effects of probenecid on ACh level in the MoTN [F_PBN_(1,5) = 1.6 (*p* > 0.1), F_event_(1,5) = 16.1 (*p* < 0.01), F_PBN*event_(1,5) = 0.1 (*p* > 0.1)], STN [F_PBN_(1,5) = 0.3 (*p* > 0.1), F_event_(1,5) = 12.0 (*p* < 0.01), F_PBN*event_(1,5) = 0.1 (*p* > 0.1)] and SNr [F_PBN_(1,5) = 1.0 (*p* > 0.1), F_event_(1,5) = 17.1 (*p* < 0.01), F_PBN*event_(1,5) = 0.2 (*p* > 0.1)]. F-values of effects of carbenoxolone on ACh level in the MoTN [F_CBX_(1,5) = 0.1 (*p* > 0.1), F_event_(1,5) = 30.9 (*p* < 0.01), F_CBX*event_(1,5) = 3.6 (*p* > 0.1)], STN [F_CBX_(1,5) = 0.2 (*p* > 0.1), F_event_(1,5) = 17.4 (*p* < 0.01), F_CBX*event_(1,5) = 1.2 (*p* > 0.1)] and SNr [F_CBX_(1,5) = 0.1 (*p* > 0.1), F_event_(1,5) = 9.8 (*p* < 0.01), F_CBX*event_(1,5) = 0.6 (*p* > 0.1)].

In the wild type, neither perfusion with non-selective hemichannels inhibitor, 100 μM carbenoxolone, nor relatively selective pannexin1-hemichannels inhibitor, 300 μM probenecid, into the PPN affected extracellular levels of L-glutamate and ACh during both wakefulness and SWS (Figure 5).

**Figure 6:**
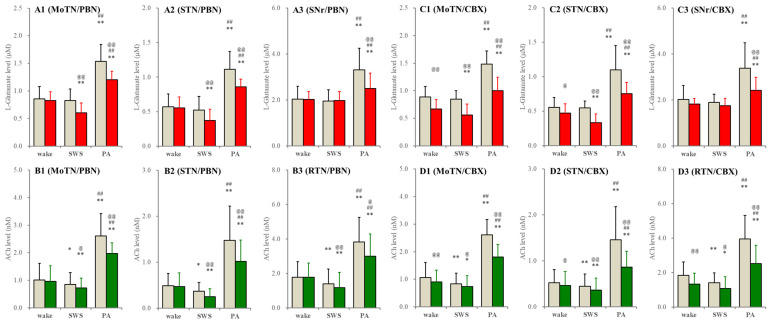
Effects of perfusion with hemichannel inhibitors into the PPN on extracellular levels of L-glutamate and ACh during wakefulness (wake), SWS and paroxysmal arousal in the MoTN, STN, SNr and RTN of S286L-TG. Panels (**A**,**B**) and (**C**,**D**) indicate the effects of perfusion with 300 μM probenecid (pannexin1-hemichannel inhibitor) and 100 μM carbenoxolone (non-selective connexin43-hemichannel inhibitor) on extracellular levels of L-glutamate (panels **A** and **C**) and ACh (panels **B** and **D**) in the MoTN (panels **A1**–**D1**), STN (panels **A2**–**D2**), SNr (panels **A3** and **C3**) and RTN (panels **B3** and **D3**) of S286L-TG during wakefulness, SWS and paroxysmal arousal (PA). Ordinates indicate the mean ± SD (*n* = 6) of extracellular levels of ACh (nM) and L-glutamate (μM). Grey columns indicate the levels of control (perfusion with MRS alone). Red and green columns indicate the levels of L-glutamate and ACh during the perfusion with MRS containing probenecid or carbenoxolone, respectively. * *p* < 0.05, ** *p* < 0.01 relative to wakefulness, @ *p* < 0.05, @@ *p* < 0.01 relative to control ## *p* < 0.01 relative to SWS using MANOVA with Scheffe’s post hoc test. F-values of effects of probenecid on L-glutamate level in the MoTN [F_PBN_(1,5) = 40.3 (*p* < 0.01), F_event_(2,10) = 249.9 (*p* < 0.01), F_PBN*event_(2,10) = 9.1 (*p* < 0.01)], STN [F_PBN_(1,5) = 15.8 (*p* < 0.01), F_event_(2,10) = 100.2 (*p* < 0.01), F_PBN*event_(2,10) = 12.4 (*p* < 0.01)] and SNr [F_PBN_(1,5) = 4.6 (*p* > 0.05), F_event_(2,10) = 20.4 (*p* < 0.01), F_PBN*event_(2,10) = 31.4 (*p* < 0.01)]. F-values of effects of carbenoxolone on L-glutamate level in the MoTN [F_CBX_(1,5) = 122.3 (*p* < 0.01), F_event_(2,20) = 121.2 (*p* < 0.01), F_CBX*event_(2,20) = 24.9 (*p* < 0.1)], STN [F_CBX_(1,5) = 43.9 (*p* < 0.01), F_event_(2,20) = 50.9 (*p* < 0.01), F_CBX*event_(2,20) = 3.3 (*p* > 0.05)] and SNr [F_CBX_(1,5) = 10.5 (*p* < 0.01), F_event_(2,20) = 25.6(*p* < 0.01), F_CBX*event_(2,20) = 9.5 (*p* < 0.01)]. F-values of effects of probenecid on ACh level in the MoTN [F_PBN_(1,5) = 6.8 (*p* < 0.05), F_event_(2,10) = 116.8 (*p* < 0.01), F_PBN*event_(2,10) = 4.4 (*p* < 0.05)], STN [F_PBN_(1,5) = 23.4 (*p* < 0.01), F_event_(2,10) = 24.3(*p* < 0.01), F_PBN*event_(2,10) = 8.7 (*p* < 0.01)] and RTN [F_PBN_(1,5) = 16.3 (*p* < 0.01), F_event_(2,10) = 67.7 (*p* < 0.01), F_PBN*event_(2,10) = 9.8 (*p* < 0.01)]. F-values of effects of carbenoxolone on ACh level in the MoTN [F_CBX_(1,5) = 47.9 (*p* < 0.01), F_event_(2,20) = 32.6 (*p* < 0.01), F_CBX*event_(2,20) = 11.5 (*p* < 0.1)], STN [F_CBX_(1,5) = 15.6 (*p* < 0.05), F_event_(2,20) = 41.8 (*p* < 0.01), F_CBX*event_(2,20) = 7.4 (*p* < 0.05)] and RTN [F_CBX_(1,5) = 21.3 (*p* < 0.01), F_event_(2,20) = 39.0 (*p* < 0.01), F_CBX*event_(2,20) = 16.8 (*p* < 0.01)].

In S286L-TG, perfusion of carbenoxolone into the PPN decreased L-glutamate levels in the MoTN, STN and SNr during both wakefulness and SWS. Probenecid decreased L-glutamate levels in the MoTN and STN during SWS but did not affect those during wakefulness (Figure 6). Neither probenecid nor carbenoxolone affected L-glutamate levels in the SNr during wakefulness and SWS. Carbenoxolone decreased ACh levels in the MoTN, STN and RTN during both wakefulness and SWS. Probenecid decreased ACh levels in the MoTN, STN and RTN during SWS but did not affect them during wakefulness (Figure 6). However, increasing releases of ACh and L-glutamate during paroxysmal arousal in all experimental regions were suppressed by perfusion with carbenoxolone and probenecid into the PPN (Figure 6). These results suggest that neither the connexin43 hemichannel nor the pannexin1 hemichannel in the PPN contribute to glutamatergic and AChergic tripartite synaptic transmission in networks among the MoTN, STN and SNr in the wild type; however, these hemichannels in the S286L-TG PPN also do not contribute to glutamatergic and AChergic transmission in networks among the MoTN, STN and SNr during wakefulness but activate them during SWS.

### 2.3. Effects of Perfusion with Inhibitors of NMDAR and AMPAR into the PPN on Extracellular Levels of ACh and L-Glutamate During Wakefulness, SWS and Interictal Discharge

**Figure 7:**
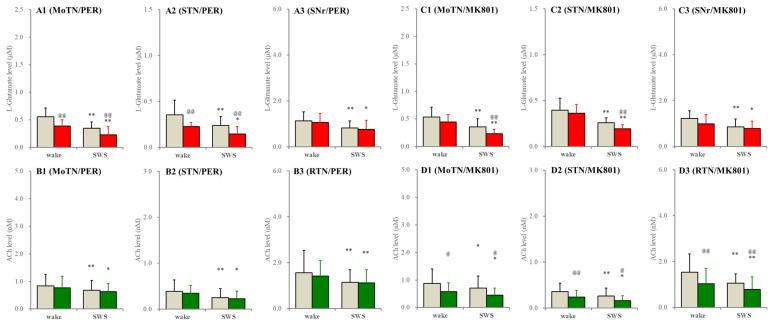
Effects of perfusion with inhibitors of AMPAR and NMDAR into the PPN on extracellular levels of L-glutamate and ACh during wakefulness (wake) and SWS in the MoTN, STN, SNr and RTN of the wild type. Panels (**A**,**B**) and (**C**,**D**) indicate the effects of perfusion with 3 μM perampanel (selective AMPAR inhibitor) and 3 μM MK801 (selective NMDAR inhibitor) on extracellular levels of L-glutamate (panels **A** and **C**) and ACh (panels **B** and **D**) in the MoTN (panels **A1**–**D1**), STN (panels **A2**–**D2**), SNr (panels **A3** and **C3**) and RTN (panels **B3** and **D3**) of the wild type during wakefulness and SWS. Ordinates indicate the mean ± SD (*n* = 6) of extracellular levels of ACh (nM) and L-glutamate (μM). Grey columns indicate the levels of control (perfusion with MRS alone). Red and green columns indicate the levels of L-glutamate and ACh during the perfusion with MRS containing probenecid or carbenoxolone, respectively. * *p* < 0.05, ** *p* < 0.01 relative to wakefulness, @ *p* < 0.05, @@ *p* < 0.01 relative to control (inhibitor free) using MANOVA with Scheffe’s post hoc test. F-values of effects of perampanel on L-glutamate level in the MoTN [F_PER_(1,5) = 54.5 (*p* < 0.01), F_event_(1,5) = 157.4 (*p* < 0.01), F_PER*event_(1,5) = 1.3 (*p* > 0.1)], STN [F_PER_(1,5) = 18.8 (*p* < 0.01), F_event_(1,5) = 19.7 (*p* < 0.01), F_PER*event_(1,5) = 0.3 (*p* > 0.1)] and SNr [F_PER_(1,5) = 11.6 (*p* < 0.01), F_event_(1,5) = 36.0 (*p* < 0.01), F_PER*event_(1,5) = 0.1 (*p* > 0.1)]. F-values of effects of MK801 on L-glutamate level in the MoTN [F_MK801_(1,5) = 17.1 (*p* < 0.01), F_event_(1,5) = 37.3 (*p* < 0.01), F_MK801*event_(1,5) = 2.4 (*p* > 0.1)], STN [F_MK801_(1,5) = 3.5 (*p* > 0.1), F_event_(1,5) = 32.3 (*p* < 0.01), F_MK801*event_(1,5) = 2.4 (*p* > 0.1)] and SNr [F_MK801_(1,5) = 1.9 (*p* > 0.1), F_event_(1,5) = 14.5 (*p* < 0.01), F_MK801*event_(1,5) = 0.6 (*p* > 0.1)]. F-values of effects of perampanel on ACh level in the MoTN [F_PER_(1,5) = 3.9 (*p* > 0.1), F_event_(1,5) = 7.1 (*p* < 0.05), F_PER*event_(1,5) = 0.1 (*p* > 0.1)], STN [F_PER_(1,5) = 2.1 (*p* > 0.1), F_event_(1,5) = 21.1 (*p* < 0.01), F_PER*event_(1,5) = 0.2 (*p* > 0.1)] and RTN [F_PER_(1,5) = 0.6 (*p* > 0.1), F_event_(1,5) = 8.0 (*p* < 0.01), F_PER*event_(1,5) = 0.4 (*p* > 0.1)]. F-values of effects of MK801 on ACh level in the MoTN [F_MK801_(1,5) = 6.7 (*p* < 0.05), F_event_(1,5) = 9.1 (*p* < 0.05), F_MK801*event_(1,5) = 9.2 (*p* < 0.05)], STN [F_MK801_(1,5) = 17.9 (*p* < 0.01), F_event_(1,5) = 13.4 (*p* < 0.01), F_MK801*event_(1,5) = 0.4 (*p* > 0.1)] and RTN [F_MK801_(1,5) = 8.2 (*p* < 0.05), F_event_(1,5) = 6.9 (*p* < 0.05), F_MK801*event_(1,5) = 0.3 (*p* > 0.1)].

In the wild type, perfusion with selective AMPAR inhibitor, 3 μM perampanel, into the PPN decreased extracellular L-glutamate levels during both wakefulness and SWS, whereas perfusion with selective NMDAR inhibitor, 3 μM MK801, into the PPN decreased L-glutamate levels during the SWS but did not affect those during wakefulness in the MoTN and STN. L-glutamate level in the SNr was unaffected by perampanel or MK801. MK801 decreased ACh levels during wakefulness and SWS, whereas perampanel did not affect them (Figure 7).

**Figure 8:**
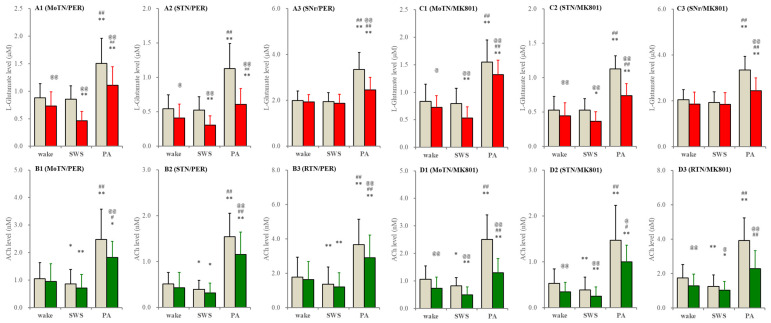
Effects of perfusion with inhibitors of AMPAR and NMDAR into the PPN on extracellular levels of L-glutamate and ACh during wakefulness, SWS and paroxysmal arousal in the MoTN, STN, SNr and RTN of S286L-TG. Panels (**A**,**B**) and (**C**,**D**) indicate the effects of perfusion with 3 μM perampanel (AMPAR inhibitor) and 3 μM MK801 (NMDAR inhibitor) on extracellular levels of L-glutamate (panels **A** and **C**) and ACh (panels **B** and **D**) in the MoTN (panels **A1**–**D1**), STN (panels **A2**–**D2**), SNr (panels **A3** and **C3**) and RTN (panels **B3** and **D3**) of S286L-TG during wakefulness, SWS and paroxysmal arousal (PA). Ordinates indicate the mean ± SD (*n* = 6) of extracellular levels of ACh (nM) and L-glutamate (μM). Grey columns indicate the levels of control (perfusion with MRS alone). Red and green columns indicate the levels of L-glutamate and ACh during the perfusion with MRS containing perampanel or MK801, respectively. * *p* < 0.05, ** *p* < 0.01 relative to wakefulness, @ *p* < 0.05, @@ *p* < 0.01 relative to control and # *p* < 0.05, ## *p* < 0.01 relative to SWS using MANOVA with Scheffe’s post hoc test. F-values of effects of perampanel on L-glutamate level in the MoTN [F_PER_(1,5) = 59.5 (*p* < 0.01), F_event_(2,10) = 65.3 (*p* < 0.01), F_PER*event_(2,10) = 14.2 (*p* < 0.01)], STN [F_PER_(1,5) = 115.0 (*p* < 0.01), F_event_(2,10) = 54.0 (*p* < 0.01), F_PER*event_(2,10) = 6.8 (*p* < 0.05)] and SNr [F_PER_(1,5) = 38.7 (*p* < 0.01), F_event_(2,10) = 58.1 (*p* < 0.01), F_PER*event_(2,10) = 23.0 (*p* < 0.01)]. F-values of effects of MK801 on L-glutamate level in the MoTN [F_MK801_(1,5) = 18.3 (*p* < 0.01), F_event_(2,20) = 135.9 (*p* < 0.01), F_MK801*event_(2,20) = 2.5 (*p* > 0.1)], STN [F_MK801_(1,5) = 52.8 (*p* < 0.01), F_event_(2,20) = 220.0 (*p* < 0.01), F_MK801*event_(2,20) = 23.7 (*p* < 0.01)] and SNr [F_MK801_(1,5) = 9.6 (*p* < 0.05), F_event_(2,20) = 17.0 (*p* < 0.01), F_MK801*event_(2,20) = 3.6 (*p* > 0.1)]. F-values of effects of perampanel on ACh level in the MoTN [F_PER_(1,5) = 8.3 (*p* < 0.05), F_event_(2,10) = 60.5 (*p* < 0.01), F_PER*event_(2,10) = 4.3 (*p* < 0.05)], STUN [F_PER_(1,5) = 19.6 (*p* < 0.01), F_event_(2,10) = 57.4 (*p* < 0.01), F_PER*event_(2,10) = 20.2 (*p* < 0.01)] and RTN [F_PER_(1,5) = 19.5 (*p* < 0.01), F_event_(2,10) = 64.5 (*p* < 0.01), F_PER*event_(2,10) = 9.8 (*p* < 0.01)]. F-values of effects of MK801 on ACh level in the MoTN [F_MK801_(1,5) = 50.1 (*p* < 0.01), F_event_(2,20) = 57.8 (*p* < 0.01), F_MK801*event_(2,20) = 17.2 (*p* < 0.01)], STN [F_MK801_(1,5) = 6.8 (*p* < 0.05), F_event_(2,20) = 45.5 (*p* < 0.01), F_MK801*event_(2,20) = 1.9 (*p* > 0.1)] and RTN [F_MK801_(1,5) = 61.0 (*p* < 0.01), F_event_(2,20) = 57.3 (*p* < 0.01), F_MK801*event_(2,20) = 30.1(*p* < 0.01)].

In S286L-TG, perfusion with MK801 and perampanel into the PPN decreased extracellular L-glutamate levels in the MoTN and STN during wakefulness and SWS but did not affect those in the SNr (Figure 8). MK801 decreased ACh level in the MoTN, STN and RTN during wakefulness and SWS, whereas perampanel did not affect them (Figure 8). Drastically increasing releases of ACh and L-glutamate during paroxysmal arousal in all experimental regions were decreased by perfusion with MK801 and perampanel into the PPN (Figure 8).

### 2.4. Fluctuation of Extracellular Levels of ACh and L-Glutamate Before and After Nocturnal Paroxysmal Dystonia

**Figure 9:**
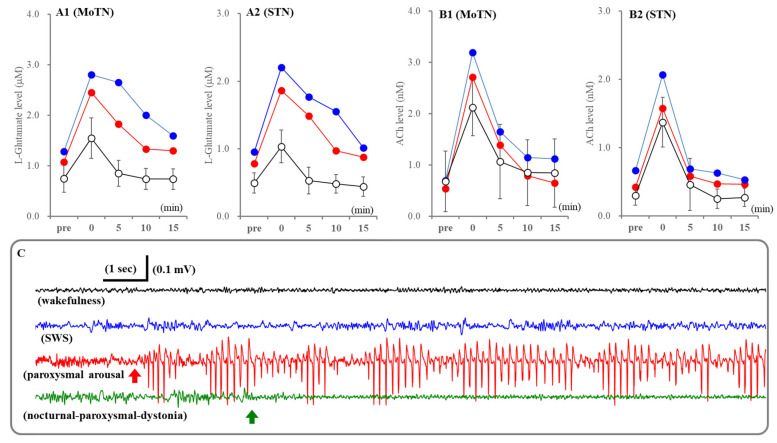
Fluctuations of extracellular levels L-glutamate and ACh in the MoTN and STN before and after paroxysmal arousal and nocturnal paroxysmal dystonia. Panels A and B indicate the extracellular levels of L-glutamate and ACh in the MoTN (panels **A1** and **B1**) and STN (panels **A2** and **B2**) of S286L-TG before and after paroxysmal arousal (opened circles) and nocturnal paroxysmal dystonia (red and blue circles). Ordinates indicate the mean ± SD (*n* = 6) of extracellular levels of ACh (nM) and L-glutamate (μM) associated with paroxysmal arousal (*n* = 6) and those of individuals associated with nocturnal paroxysmal dystonia. Abscissas indicate before (pre) and after the paroxysmal arousal or nocturnal paroxysmal dystonia (min). Panel (**C**) indicates the typical ECoG during wakefulness (black), SWS (blue), paroxysmal arousal (red) and nocturnal paroxysmal dystonia (green). Red and green arrows indicate the onsets of paroxysmal arousal and nocturnal paroxysmal dystonia, respectively.

In this study, extracellular levels of ACh and L-glutamate in the MoTN and STN during before and after nocturnal paroxysmal dystonia, which was electrocorticogram (ECoG)-insensitive, could be determined in only two cases by chance (Figure 9). Therefore, neither statistical nor pharmacological analyses could be implemented. In spite of ECoG-insensitivity, extracellular levels of ACh and L-glutamate in the MoTN and STN drastically increased during nocturnal paroxysmal dystonia (Figure 9). ACh releases during nocturnal paroxysmal dystonia appeared to be equivalent to during paroxysmal arousal. L-glutamate releases during nocturnal paroxysmal dystonia were larger than during paroxysmal arousal, and increased L-glutamate after nocturnal paroxysmal dystonia persisted (Figure 9).

## 3. Discussion

**Figure 10:**
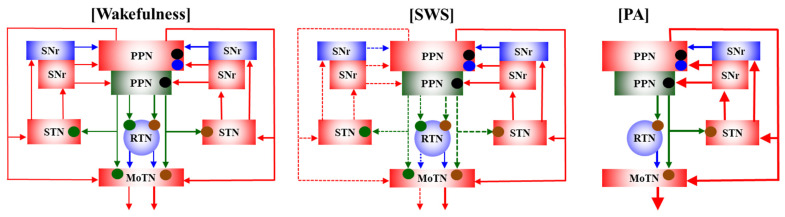
Proposed hypothesis for functional abnormalities among the PPN and its projection regions in S286L-TG. The AChergic neurons in the PPN project to the thalamus (RTN and MoTN) and STN [21,22], and glutamatergic neurons project mainly to the MoTN and STN [23,24]. GABAergic neurons in the RTN, which receives AChergic terminals via α4β2-nAChRs [10,11,16], project to various glutamatergic neurons in the thalamus [26]. SNr receives mainly glutamatergic terminals from the STN [31] and projects both GABAergic and glutamatergic terminals to the PPN [27,28,29]. In the PPN, glutamatergic neurons receive glutamatergic terminals via both AMPAR and NMDAR, whereas AChergic neurons receive mainly NMDAR. During wakefulness, glutamatergic transmissions from the PPN to its projection regions of S286L-TG are enhanced compared to the wild type, whereas AChergic transmission between S286L-TG and the wild type is almost equal. During SWS, in the wild type, both AChergic and glutamatergic transmissions from the PPN to its projection regions decrease compared to wakefulness. In S286L-TG, AChergic transmission from the PPN to its projection regions also decreases compared to wakefulness, whereas that of glutamate does not change between wakefulness and SWS. During PA (sudden/brief awakening with polyspikes), both AChergic and glutamatergic transmissions drastically increase compared to wakefulness and SWS, which are suppressed by inhibition of hemichannels, NMDAR and AMPAR in the PPN. Glutamatergic transmission from the PPN to its projection regions is suppressed by inhibition of both AMPAR and NMDAR in the PPN, whereas AChergic transmission is predominantly suppressed by inhibition of NMDAR in the PPN. Hemichannel inhibitor decreases both AChergic and glutamatergic transmissions in S286L-TG but does not affect those in the wild type. Hemichannel-dependent releases of ACh and L-glutamate during SWS are larger than those during wakefulness in S286L-TG.

This study elucidated several functional abnormalities of AChergic and glutamatergic transmissions among the PPN, thalamus (RTN and MoTN), STN and SNr that are possibly associated with the epileptogenesis/ictogenesis of S286L-TG. Based on the results, candidate transmission abnormalities among the PPN, MoTN, RTN, STN and SNr underlying paroxysmal arousal in S286L-TG are represented in Figure 10. (1) Releases of ACh and L-glutamate during polyspikes accompanying arousal (paroxysmal arousal) drastically increased and related to the activation of the hemichannel, NMDAR and AMPAR. (2) Both extracellular L-glutamate levels in the PPN and its projection regions of S286L-TG were consistently larger than the wild type, whereas ACh levels were almost equal. (3) Decreasing ACh releases accompanying SWS was maintained, but decreasing L-glutamate was impaired in S286L-TG. (4) Enhanced glutamatergic transmissions in the PPN projection regions of S286L-TG during both wakefulness and SWS were suppressed by inhibitions of hemichannel, NMDAR and AMPAR in the PPN. (5) AChergic transmissions in the PPN projection regions during wakefulness and SWS of both the wild type and S286L-TG were regulated by NMDAR. (6) Drastically increasing releases of ACh and L-glutamate induced by paroxysmal arousal were inhibited by inhibition of hemichannels, NMDAR and AMPAR in the PPN. These results suggest that enhanced hemichannel function in the PPN plays important roles in epileptogenesis/ictogenesis of paroxysmal arousal in S286L-TG via hyperactivation of both AChergic and glutamatergic transmissions among the PPN and its projection regions.

ADSHE/SHE was proposed to be defined as “Brief focal motor seizure with hyperkinetic or asymmetric tonic/dystonic features occurring predominantly during sleep” [2,7]. Focal hypermotor seizures occurring during SWS are considered to predominantly originate from the frontal cortex [2,3,8]. However, patients with ADSHE/SHE usually display various seizures, not only complex hypermotor seizures but also frequent short minormotor events and/or paroxysmal arousal in the same night [2,3,7,8]. Paroxysmal arousal is characterized by sudden/brief arousals irrespectively followed with or without motor events [2,3,7,8]. Paroxysmal arousal is considered to be a distinctive feature of ADSHE among focal seizures, since most limbic seizures are commonly associated with loss of consciousness [38]. Clinically, stereo-EEG detected that all paroxysmal arousal (including similar to normal awakenings) occurred during SWS and related to polyspikes [9,47]. Thus, targeting the phenotype of S286L-TG in this study, eye opening accompanying polyspikes during SWS resembled the clinical features of paroxysmal arousal. On the other hand, in this study, nocturnal paroxysmal dystonia involved obviously dystonic postures accompanying transformed ECoG patterns from SWS to wakefulness without detectable epileptic discharges. This phenotype of S286L-TG may be similar to clinically EEG-insensitive nocturnal paroxysmal dystonia [9,47].

Limbic seizure models demonstrated decreasing firing of AChergic and glutamatergic neurons in the PPN and thalamus during impaired consciousness/awareness with limbic seizures, whereas, conversely, stimulation of the PPN projection regions rescued consciousness during limbic seizures [42,48,49,50]. Thus, increasing releases of ACh and L-glutamate in the PPN and its projection regions during paroxysmal arousal of S286L-TG is possibly involved in the occurring arousals accompanying polyspikes. Although perfusion with hemichannel inhibitors into the PPN did not affect extracellular levels of ACh or L-glutamate in the PPN/projection regions of the wild type during wakefulness and SWS, increasing releases of ACh and L-glutamate during paroxysmal arousal were suppressed by inhibition of the hemichannel, NMDAR and AMPAR in the PPN of S286L-TG. Therefore, AChergic and glutamatergic transmissions in the PPN and its projection regions are considered to be overdriven via hyperactivation of glutamatergic tripartite synaptic transmission under paroxysmal arousal, obviously distinct from the regulation mechanism of physiological transmission associated with the sleep–wake cycle. In spite of the ECoG-insensitivity, releases of ACh and glutamate in the MoTN and STN during nocturnal paroxysmal dystonia also drastically increased. Although neither statistical nor pharmacological analyses could be implemented, due to only two cases being collected, ACh releases induced by paroxysmal arousal and nocturnal paroxysmal dystonia were almost equal and rapidly recovered to values before paroxysmal arousal and nocturnal paroxysmal dystonia, whereas increasing L-glutamate releases during nocturnal paroxysmal dystonia were larger than during paroxysmal arousal and persisted after nocturnal paroxysmal dystonia compared to paroxysmal arousal. Therefore, activation of both AChergic and glutamatergic transmissions contributes to arousals, but motor events in nocturnal paroxysmal dystonia probably require greater activation of glutamatergic transmission in the PPN/projection regions compared to paroxysmal arousal. Indeed, activation of glutamatergic PPN neurons leads to wakefulness and locomotion [23,51], whereas AChergic PPN neurons display maximal activation during wakefulness and SWS, but does not contribute to initiation of locomotion [52,53]. Thus, the discrepancy in ECoG patterns between paroxysmal arousal and nocturnal paroxysmal dystonia suggests that the propagation of epileptic discharges to the cortex is not essential, but, rather, hyperactivation of glutamatergic transmissions in the networks from the PPN to its projection regions (MoTN, STN and/or SNr) plays important roles in the generation of nocturnal paroxysmal dystonia in S286L-TG.

Onsets of polyspikes with hypermotor seizures and without motor events in both S284L-TG and S286L-TG were at approximately 8 weeks and 6 weeks of ages, respectively [1,10,12,33]. The typical frequency of HFO prior to polyspikes occurring without motor events and hypermotor seizures were ranged lower and over 250-Hz, respectively [12]. During the critical period for onset of the ADSHE hypermotor seizure in S286L-TG, both expression and function of the connexin43 hemichannel and pannexin1 hemichannel increased; however, any functional abnormalities of the primary cultured astrocytes from neonatal S286L-TG and the wild type could not be detected [1,12,17,18]. Nevertheless, artificial HFO-evoked stimulations time- and frequency-dependently increased the expression and function of the astroglial hemichannels [1,12,17,18]. Based on these previous findings, we estimated that HFO during SWS played important roles in the development of the epileptogenesis/ictogenesis of S286L-TG [1].

In the interictal stages, glutamatergic transmission among the PPN and its projection regions of S286L-TG consistently increased compared to the wild type. Additionally, the function of hemichannels in S286L-TG also consistently increased compared to the wild type during both wakefulness and SWS, since hemichannel inhibitors did not affect L-glutamate release in the wild type but suppressed those in S286L-TG. Increasing expressions of the connexin43 hemichannel and pannexin1 hemichannel are probably involved in the consistently increased L-glutamate release in S286L-TG compared to the wild type. Furthermore, comparison between during wakefulness and SWS in S286L-TG suggests that the contribution of pannexin1 hemichannels to L-glutamate release during SWS was greater than during wakefulness. Considering previous findings, these results suggest that the increased pannexin1 hemichannel in the PPN of S286L-TG is activated during SWS via possibly physiological ripple-bursts HFO (lower 250 Hz) [54]. Contrary to L-glutamate, the ACh releases of both the wild type and S286L-TG were unaffected by hemichannel inhibitors during either wakefulness or SWS; however, increasing ACh release during paroxysmal arousal was drastically suppressed by hemichannel inhibitors. Therefore, ripple-bursts HFO during SWS cannot affect ACh releases in the PPN/projection regions, whereas fast ripple-bursts HFO (over 250-Hz) in the initiation of polyspikes or paroxysmal arousal probably contributes to activation of ACh neurons in the PPN and its projections [12].

Drastically increasing extracellular L-glutamate levels in the PPN/projection regions during paroxysmal arousal can be interpreted as direct detection of released L-glutamate via activated hemichannels; however, the increasing ACh level is considered to be not released from astrocytes but rather increased release from neurons, which is indirectly modulated by astroglial function [55]. In other words, increasing AChergic transmissions in the PPN/projections during paroxysmal arousal may be activated by NMDAR and AMPAR via increasing L-glutamate release from astrocytes. Indeed, increasing ACh release during paroxysmal arousal was suppressed by inhibitors of hemichannels, NMDAR and AMPAR; however, ACh releases in the PPN/projections of both the wild type and S286L-TG during wakefulness and SWS were regulated by NMDAR but not by AMPAR or hemichannels. The discrepancy in the regulation mechanisms of ACh releases among wakefulness, SWS and paroxysmal arousal in the PPN/projections suggest the possibility that, under physiological conditions, ACh release is regulated by neurotransmission via NMDAR, but drastically increased glutamate release through hyperactivated hemichannels by paroxysmal arousal enhances ACh releases via activations of both AMPAR and NMDAR.

This hypothesis can also provide a reasonable explanation regarding the fact that decreasing releases of L-glutamate and ACh accompanying SWS were impaired and maintained in S286L-TG, respectively. Impaired decreasing L-glutamate release accompanying SWS compared to wakefulness had already been confirmed in the frontal cortex in S284L-TG [33]. Neurons in the PPN project glutamatergic and AChergic terminals to the frontal cortex, which regulate cortical arousal and behavior selection via convey sensory information [56,57]. Both expressions and functions of the connexin43 hemichannel and pannexin1 hemichannel increased in the frontal cortex of S286L-TG [12,16,17,18]. Therefore, in the regions where the function of hemichannels is increased, the physiological fluctuations of extracellular L-glutamate levels appear to disappear. However, in the interictal stage, except for epileptic discharge occurring, the physiological neuronal transmission is a possible function, since, under the inhibition of the pannexin1 hemichannel, the decreasing L-glutamate release in the MoTN and STN accompanying SWS was observed. Therefore, although the neurotransmission-dependent decreasing L-glutamate release accompanying SWS was maintained, conversely, L-glutamate release through activated hemichannel by HFO during SWS was increased, resulting in the amount of extracellular L-glutamate level seeming to be unchanged during SWS.

This study explored abnormalities in the PPN and its projection regions of AChergic and glutamatergic transmissions during wakefulness, SWS and paroxysmal arousal using microdialysis. There was a limitation in this study. Decreased ACh release in S286L-TG was maintained, whereas the consistently increasing ACh release in S286L-TG compared to the wild type could not be detected as different from L-glutamate. Actual fluctuations of ACh release possibly could not be detected due to rapidly degradation of released ACh in the extracellular spaces by acetylcholinesterase/butyrylcholinesterase. Measurement of ACh release using the conventional method, perfusion with acetylcholinesterase/butyrylcholinesterase inhibitor, is a powerful tool for detecting slight fluctuations of ACh release. In this study, although perfusion with MRS containing 1 μM neostigmine could not detect the consistently increasing ACh release in S286L-TG compared to the wild type, we cannot deny the possibility that perfusion of a larger concentration of neostigmine could detect the consistently increasing ACh release in S286L-TG compared to the wild type. However, this study did not implement a further study using higher concentration of acetylcholinesterase/butyrylcholinesterase inhibitor, since over a 10-fold increasing extracellular ACh level induced by even 1 μM neostigmine may drastically disrupt transmission balances in the circuit among the PPN and its projection regions. These limitations in this study must be clarified by immunohistochemistry that determines the acetylcholinesterase/butyrylcholinesterase in the PPN. Additionally, the immunohistochemical studies which analyze the SNAREs, including synaptophysin, syntaxin, synaptobrevin and synaptotagmin, which are exocytosis-regulating molecules, also must clarify the abnormalities in releases of L-glutamate and ACh in the networks from the PPN to its projection regions.

## 4. Materials and Methods

### 4.1. Experimental Animals

All experimental procedures, including animal care and protocols for animal experiments, were approved by the Animal Research Ethics Committee of the Mie University School of Medicine (No. 24-37-R3, 7 March 2018) and performed in accordance with the ethical guidelines established by the Institutional Animal Care and Use Committee at Mie University, Japan, and the Animal Research: Reporting of In Vivo Experiments guidelines [58].

A total of 120 male rats; S286L-TG (*n* = 60) [10] and wild-type littermates (*n* = 60) (Sprague Dawley strain background, SLC, Shizuoka, Japan) were housed individually in cages and kept in air-conditioned rooms (temperature, 22 ± 2 °C) with 12 h light/dark cycle, with ad libitum access to food and water.

### 4.2. Chemical Agents

Selective inhibitors of AMPA/glutamate receptor (AMPAR), perampanel [59,60], NMDA/glutamate receptor (NMDAR), MK801 [61,62], hemichannels containing connexin43 (connexin43 hemichannels), carbenoxolone [63,64], pannexin1 hemichannels, probenecid [63,65] and voltage-sensitive Na^+^ channel, tetrodotoxin [66,67] were obtained from Funakoshi (Tokyo, Japan). All compounds were prepared on the day of the experiment. Perampanel and probenecid were initially prepared as 10 mM and 50 mM in dimethyl sulfoxide, respectively. Carbenoxolone, MK801 and tetrodotoxin were dissolved directly in the experimental medium.

### 4.3. Preparation of the Microdialysis System

The rats were anesthetized with 1.8% isoflurane and placed on a stereotaxic frame [10]. Concentric direct-insertion-type dialysis probes (0.22 mm diameter, 1 mm exposed membrane: Eicom, Kyoto, Japan) were implanted in the PPN (A = −7.8 mm, L = +2.0 mm, V = −7.8 mm, relative to bregma), substantia nigra pars reticulata (SNr) (A = −5.6 mm, L = −2.3 mm, V = −8.4 mm, relative to bregma) and the subthalamic nucleus (STN) (A = −3.6 mm, L = −2.5 mm, V = −8.4 mm, relative to bregma). Another concentric direct-insertion-type probe with longer exposed membrane (0.22 mm diameter, 2 mm exposed membrane: Eicom) was implanted in the motor thalamic nuclei (MoTN), comprising ventroanterior and ventrolateral thalamic nuclei (A = −2.0 mm, L = +1.4 mm, V = −7.2 mm, relative to bregma), and reticular thalamic nucleus (RTN) (A = −1.4 mm, L = 1.2 mm, V = −7.2 mm). Recording and reference screw electrodes were fastened to the skull over the frontal (A = +2.7 mm, L = +1.8 mm, relative to bregma) and occipital (A = −10.0 mm, L = +1.8 mm, relative to bregma) region. Electrocorticograms (ECoG) of the freely moving rats were telemetrically recorded (Unimec, Tokyo, Japan) and analyzed using PowerLab (AD Instruments, Dunedin, New Zealand). The location of the dialysis probe was verified at the end of each experiment using 300 μm thick brain tissue slices (Vibratome 1000, Technical Products International, St. Louis, MO, USA).

Perfusion experiments began 18 h after recovery from anesthesia at a constant rate of 2 μL/min with modified Ringer solution (MRS: 145 Na^+^, 2.7 K^+^, 1.2 Ca^2+^, 1.0 Mg^2+^ and 154.4 Cl^−^, buffered at pH 7.4, with 2 mM phosphate buffer and 1.1 mM Tris buffer) [10,11]. Extracellular transmitter levels were measured 8 hr after starting perfusion. Dialysates were every collected 5 min interval. According to ECoG, the dialysates were classified into periods of wakefulness, SWS and paroxysmal arousal (lasting over 10 s spike complexes). To study the impacts of inhibitors of hemichannels, NMDAR and AMPAR on extracellular transmitter levels, MRS containing 100 μM carbenoxolone, 300 μM probenecid, 3 μM MK801 or 3 μM perampanel was perfused into the PPN. Each dialysate was injected into an ultra-high-pressure liquid chromatograph (UHPLC) for determination of levels of ACh, GABA and L-glutamate.

### 4.4. Determination of Levels of ACh, GABA and L-Glutamate

Levels of GABA and L-glutamate were determined using UHPLC (PU-4185, Jasco, Tokyo, Japan) with fluorescence resonance energy transfer detection (FP-4020, Jasco), after dual derivatization with isobutyryl-L-cysteine and o-phthalaldehyde. Derivatized samples (5 μL) were injected using an autosampler (AS-4150, Jasco). An analytical column (Triat C18, particle 1.8 µm, 50 × 2.1 mm, YMC, Kyoto, Japan) was maintained at 45 °C, with flow rate set at 500 μL/min. A linear gradient elution program was performed over 10 min with mobile phases A (0.05 M citrate buffer, pH 5.0) and B (0.05 M citrate buffer containing 30% acetonitrile and 30% methanol, pH 3.5). The excitation/emission wavelengths of the fluorescence detector were set at 345/455 nm.

ACh level was analyzed using UHPLC equipped with mass spectrometry (LCMS) (Acquity H-Class equipped with an Acquity SQ detector; Waters, Milford, MA, USA). Samples (5 μL aliquots) were automatically injected into an autosampler (Acquity Sample Manager FTN; Waters) and separated using a graphite carbon column (particle size: 3 μm, 150 × 2.1 mm; Hypercarb, Thermo, Waltham, MA, USA) maintained at 400 μL/min at 50 °C. The LCMS procedure for ACh was as follows. A linear gradient elution program was used for more than 10 min with mobile phases A (1 mM acetate) and B (100% acetonitrile). Nitrogen flow rates of desolvation and cone were set at 750 L/h and 5 L/h, respectively. The temperature for desolvation was set at 450 °C. The cone voltage for the measurement of ACh (*m*/*z* = 146) was 25 V.

### 4.5. Capillary Immunoblotting

To analyze the expression of connexin43 and pannexin1 in the PPN, the PPN was extracted using the Minute Plasma Membrane Protein Isolation Kit (Invent Biotechnologies, Plymouth, MN, USA). Capillary immunoblotting was performed using Wes (ProteinSimple, Santa Clara, CA, USA) according to the ProteinSimple user manual [10]. Plasma membrane fractions were mixed with master mix (ProteinSimple) until a final concentration of 1 × sample buffer, 1 × fluorescent molecular weight marker and 40 mM dithiothreitol was obtained, which was then heated at 95 °C for 5 min. Samples, blocking reagent, primary antibodies, horseradish peroxidase (HRP)-conjugated secondary antibody, chemiluminescent substrate (SuperSignal West Femto; Thermo) and separation/stacking matrices were also distributed into designated 25-well plates. Separation electrophoresis and immunodetection were automatedly performed in Wes. Target proteins were probed with primary antibodies, followed by incubation with HRP-conjugated secondary antibodies (anti-rabbit HRP-conjugated IgG, A00098, 10 μg/mL, GenScript, Piscataway, NJ, USA). Antibodies against GAPDH (NB300-327, 1:500, Novus Biologicals, Littleton, CO, USA), connexin43 (C6219, 1:100, Sigma-Aldrich, St. Louis, MO, USA) [68] and pannexin1 (12595-1-AP, 1:100, Proteintech, Rosemont, IL, USA) [69,70] were diluted in Immuno Shot Platinum (CosmoBio, Tokyo, Japan).

### 4.6. Data Analysis

All experiments were designed with groups containing equal numbers of animals (*n* = 6), without formal power analysis, according to previous studies. All values were expressed as the mean ± SD, and *p*-values < 0.05 were considered statistically significant. The levels of drugs for administration were selected based on previous reports. To determine the levels of ACh, L-glutamate, GABA and protein, the sample order of the autosamplers were set using random-number tables, as this was possible as blinding.

To compare the changes in extracellular levels of ACh, GABA and L-glutamate in the PPN, RTN, MoTN, STN and SNr of the individuals among wakefulness, SWS and paroxysmal arousal, extracellular levels were analyzed by multivariate analysis of variance (MANOVA) with Scheffe’s post hoc test using BellCurve for Excel version 3.2 (Social Survey Research Information, Tokyo, Japan). When the data did not violate the assumption of sphericity (*p* > 0.05), the F-value of the MANOVA was analyzed using assumed degrees of freedom of sphericity. When the assumption of sphericity was violated (*p* < 0.05), the F-value was analyzed using Greenhouse–Geisser-corrected degrees of freedom. When the F-value for the factors of event (wakefulness/SWS/paroxysmal arousal), genotype (wild type/S286L-TG) or agent (carbenoxolone, probenecid, MK801, perampanel) of the MANOVA was significant, the data were analyzed using Scheffe’s post hoc test. Expressions of connexin43 and pannexin1 in the plasma membrane fraction were analyzed using Student’s *T*-test.

## 5. Conclusions

This study elucidated the functional abnormalities of glutamatergic and AChergic transmissions in S286L-TG; however, unexpectedly, glutamatergic transmission was severely impaired compared to ACh among the PPN/projections in S286L-TG. Decreasing L-glutamate release accompanying SWS was impaired, but that of ACh release was maintained in the PPN/projections of S286L-TG. In the interictal stage, extracellular L-glutamate levels of S286L-TG consistently increased compared to the wild type, but those of ACh were almost equal between the wild type and S286L-TG during both wakefulness and SWS. Increasing expression and function of the connexin43 hemichannel and pannexin1 hemichannel in the PPN of S286L-TG are involved in the predominant dysfunction of glutamatergic transmission in S286L-TG. Candidate mechanisms of these dysfunctions of glutamatergic transmissions were possibly caused by hyperactivated hemichannels, but those of AChergic transmissions were maintained. These functional abnormalities during the interictal stage shift to enhanced epileptogenic excitatory transmission imbalance, and impaired deceasing L-glutamate release accompanying SWS leads to further enhanced epileptogenic excitatory transmission. Ripple-bursts HFO during SWS contributes to further activation of the connexin43 hemichannel and pannexin1 hemichannel in the PPN, resulting in generating paroxysmal arousal (sudden/brief awakening accompanied with polyspikes). Finally, selective inhibitors of the pannexin1 hemichannel and/or connexin43 hemichannel may be attractive therapeutic targets for suppressing the development of ictogenesis with epileptogenesis in ADSHE.

## Data Availability

The raw data supporting the conclusions of this article will be made available by the authors on request.

## Abbreviations

The following abbreviations are used in this manuscript:

ADSHE	autosomal dominant sleep-related hypermotor epilepsy
AMPA	α-Amino-3-hydroxy-5-methyl-4-isoxazolepropionic acid
AMPAR	AMPA/glutamate receptor
GAPDH	glyceraldehyde 3-phosphate dehydrogenase
MoTN	motor thalamic nuclei
nAChR	nicotinic ACh receptor
NMDA	N-methyl-D-aspartic acid
NMDAR	NMDA/glutamate receptor
SWS	slow-wave sleep
PPN	pedunculopontine nucleus
RTN	reticular thalamic nucleus
SNr	substantia nigra pars reticulata
STN	subthalamic nucleus
UHPLC	ultra-high performance liquid chromatography
LCMS	UHPLC with mass spectrometry
SD	standard deviation
